# A passive dorsiflexing ankle prosthesis to increase minimum foot clearance during swing

**DOI:** 10.1017/wtc.2023.10

**Published:** 2023-05-15

**Authors:** Harrison L. Bartlett, Max K. Shepherd, Brian E. Lawson

**Affiliations:** 1 Little Room Innovations, LLC, Ann Arbor, MI, USA; 2 Department of Mechanical Engineering, Northeastern University, Boston, MA, USA; 3 Department of Physical Therapy, Northeastern University, Boston, MA, USA

**Keywords:** Amputation, biomechanics, minimum foot clearance, gait, foot, prosthesis

## Abstract

The biological ankle dorsiflexes several degrees during swing to provide adequate clearance between the foot and ground, but conventional energy storage and return (ESR) prosthetic feet remain in their neutral position, increasing the risk of toe scuffs and tripping. We present a new prosthetic ankle intended to reduce fall risk by dorsiflexing the ankle joint during swing, thereby increasing the minimum clearance between the foot and ground. Unlike previous approaches to providing swing dorsiflexion such as powered ankles or hydraulic systems with dissipative yielding in stance, our ankle device features a spring-loaded linkage that adopts a neutral angle during stance, allowing ESR, but adopts a dorsiflexed angle during swing. The ankle unit was designed, fabricated, and assessed in level ground walking trials on a unilateral transtibial prosthesis user to experimentally validate its stance and swing phase behaviors. The assessment consisted of three conditions: the ankle in an operational configuration, the ankle in a locked configuration (unable to dorsiflex), and the subject’s daily use ESR prosthesis. When the ankle was operational, minimum foot clearance (MFC) increased by 13 mm relative to the locked configuration and 15 mm relative to his daily use prosthesis. Stance phase energy return was not significantly impacted in the operational configuration. The increase in MFC provided by the passive dorsiflexing ankle prosthesis may be sufficient to decrease the rate of falls experienced by prosthesis users in the real world.

## 1. Introduction

Over 50% of lower limb prosthesis users report having fallen at least once in the past year (Kulkarni et al., 1996; Gauthier-Gagnon et al., 1999; Miller et al., 2001; Kim et al., 2019), and approximately 1/3 of fallers report injuries (Hunter et al., 2017). This high fall rate leads to a fear of falling, which can lead to activity avoidance, thereby decreasing the quality of life for this population (Miller et al., 2001). Aside from the impact that falls have on the quality of life of prosthesis users, falls also impose a substantial financial burden on both the prosthesis user and the healthcare system. Specifically, the 6-month cost associated with a fall that precipitated an emergency room visit was placed at $18,091 while the 6-month cost associated with a fall that precipitated a hospital admission was estimated at $25,652 (Mundell et al., 2017). Therefore, reducing the rate of falls within this population has the potential to improve patient quality of life while simultaneously reducing the significant financial burden associated with falls.

A recent study on the causes of falls amongst lower limb prosthesis users found that a majority of falls are a result of a trip, a slip, or a problem with the prosthesis (Kim et al., 2019). Furthermore, 22% of falls occurred due to trips while walking, which was the activity with the highest rate of falls by a factor of four (Kim et al., 2019). The same study showed that the vast majority of these falls occurred while walking on level terrain (approximately 50%) (Kim et al., 2019). In this work, a trip was defined as an unanticipated contact between the foot and an obstacle during swing phase. The obstacle could be a distinct feature, such as a crack in a sidewalk, or the ground itself (commonly the case in Kim et al., 2019). As such, to combat the high rate of falls in this population, the level ground walking behavior of the device should be examined and improved.

The current standard of care prosthetic foot is a spring-like energy storage and release (ESR) foot that is configured at a neutral (nondorsiflexed) angle during swing phase (Whiteside et al., 2007). An ESR foot is capable of storing energy from the user during early and middle stance and returning a proportion of that energy during terminal stance. Once ground contact is lost, however, the device immediately returns to its neutral configuration which poses a significant risk of a scuff or stumble when encountering an unanticipated obstacle (Ludviksdottir et al., 2013; Johnson et al., 2014; Lamers et al., 2018; Bartlett et al., 2022b).

Several studies have shown that increasing ground clearance in swing, also known as minimum foot clearance (MFC), can dramatically reduce the risk of falls (Begg et al., 2007; Khandoker et al., 2008; Rosenblatt et al., 2014; Rosenblatt et al., 2017). Specifically, in one laboratory-based study conducted on eight unilateral transtibial prosthesis users, 5° of swing phase ankle dorsiflexion was shown to decrease the likelihood of tripping over a 5 mm high, unexpected obstacle from 1 in every 166 steps to 1 in 3,169 steps (a reduction of 95% in trip risk) (Rosenblatt et al., 2014). These fall reduction results are not only limited to laboratory-based studies, but also extend to a reduction of falls in the real world. Over a 1-year period, prosthesis users who experienced zero falls exhibited an MFC that was more than 50% higher than individuals who experienced at least one trip-related fall (Rosenblatt et al., 2017).

MFC is a result of the configuration of the hip, knee, and ankle joints during the swing phase of gait (Sensinger et al., 2013. One strategy for increasing MFC is to design prosthetic knees and/or ankles that adopt a flexed configuration during swing (Bellmann et al., 2010; Sensinger et al., 2013; Lura et al., 2015; Köhler et al., 2020; Quraishi et al., 2021; Bartlett et al., 2022a), thereby increasing the clearance between the foot and ground. When comparing interventions targeted at both joints, however, ankle dorsiflexion has been shown to be a more effective strategy due to the high sensitivity of MFC to changes in ankle angle at the instant of minimum clearance (Moosabhoy and Gard, 2006; Sensinger et al., 2013. As a result, a variety of prosthetic ankles have been developed to promote swing phase dorsiflexion in both industry and academia.

One approach to providing swing phase dorsiflexion is to utilize a mechatronic system to change the configuration of the ankle while walking. Such a device requires onboard actuation to reposition the ankle joint during swing phase. The prototypical example is the Proprio Foot by Ossur, but fully powered devices such as the Empower Ankle by Ottobock also employ this strategy. The Proprio Foot, which is a lightly powered ankle capable of swing phase repositioning, has a mass of 1.43 kg and a battery life of 18 to 36 hr, depending on usage. The Proprio Foot uses its onboard actuator to dorsiflex (approximately 5°) and then plantarflex the ankle during swing phase (Rosenblatt et al., 2014; Lamers et al., 2018). Similar devices have also been developed in an academic setting and are able to achieve reliable swing phase dorsiflexion using a variety of actuation approaches (Au and Herr, 2008; Shultz et al., 2013; Lenzi et al., 2017; Bartlett et al., 2021). Implementing swing phase dorsiflexion through these approaches substantially increases the size, weight, and complexity of the prosthetic ankle (Delussu et al., 2013; Kim et al., 2021), though these devices are designed to offer additional benefits beyond swing dorsiflexion, unlike the device presented here. For a given user, these tradeoffs must be weighed against the benefits of these devices (such as a reduced fall risk or the ability to adapt to various terrains and activities), which can make the decision to adopt the device difficult when compared to other commercial alternatives.

Another approach for providing swing phase dorsiflexion is to employ a clutching mechanism. In an early stance, the ankle is forced into plantarflexion from the user’s weight, and a clutch can then lock the ankle in mid-stance. The clutch is released in swing and a compliant element is used in parallel to the clutch to return the ankle to a dorsiflexed configuration. The clutch mechanism can be either purely mechanical (often weight-activated) or electromechanical in nature (microcontroller-activated based upon sensor signals). Several research prototypes utilize this design approach (Nickel et al., 2014; Amiot et al., 2017; Holgate et al., 2017; Lee et al., 2017; Heremans et al., 2019). These clutch-based devices are typically intended to also adapt to varying ground slopes, and as such, are typically heavier and more complex than is necessary to solely achieve the swing phase dorsiflexion functionality.

A third approach to providing swing phase dorsiflexion utilizes a hydraulic actuator unit. Hydraulic ankles provide a damping-type resistance through a fluid cylinder and an orifice restriction. These devices achieve swing phase dorsiflexion by yielding in the dorsiflexion direction while subject to stance phase torques. They remain in a dorsiflexed position throughout swing phase, and the subsequent heel strike serves to plantarflex the ankle back to a nominal position. It should be noted that, in addition to providing swing phase dorsiflexion, these hydraulic devices have also shown benefits when walking and standing on sloped ground (Struchkov and Buckley, 2016; Hahn et al., 2018; Ernst et al., 2022). Several commercial prosthetic devices (such as the Elan by Blatchford) employ this approach for providing swing phase dorsiflexion (Johnson et al., 2014; Struchkov and Buckley, 2016; Hahn et al., 2018; Riveras et al., 2020; Ernst et al., 2022). However, these hydraulic prosthetic ankles dissipate energy in their damper units during stance phase, and this energy cannot be returned to the user at terminal stance. As such, these hydraulic ankles sacrifice the energy storage and return (ESR) capabilities of ESR feet to achieve swing phase dorsiflexion (Davot et al., 2021).

Based on the potential benefit to be derived from providing swing phase foot clearance and the various tradeoffs associated with the present state of prosthetic technology, there is a significant clinical need for a prosthetic ankle that provides swing phase dorsiflexion. Moreover, the device should also maintain low mass, size, and complexity while simultaneously retaining the energy storage and release properties of the standard of care ESR feet while walking on level ground. To that end, this work presents the design and biomechanical assessment of a mechanically passive prosthetic ankle that achieves both stance phase ESR as well as swing phase dorsiflexion while maintaining a small size and mass. The design concept and theory are presented and then implemented in a functional prosthetic device. The device is then assessed in level ground walking experiments on a single unilateral transtibial prosthesis user.

## 2. Design

Unlike prior works, the ankle design presented here is intended to obtain swing phase dorsiflexion as well as stance phase ESR without incorporating other biomechanical features such as slope adaptation. This narrowing of scope is leveraged to yield a compact and lightweight mechanism that achieves these biomechanical goals without the use of any electromechanical or hydraulic componentry.

The ankle mechanism is mechanically passive and dorsiflexes during swing phase. As such, it is referred to in this article as the passive dorsiflexing ankle prosthesis (PDAP).

### 2.1. Goals and design requirements

The primary goal of the prosthetic ankle joint presented here is to increase MFC in swing phase without sacrificing the stance phase energy storage and release behavior of standard ESR feet. Furthermore, the goal was to achieve this combination of behaviors in a form factor that minimizes size, mass, and complexity while maintaining durability. To this end, a set of design guidelines and requirements was drafted.

A target swing phase dorsiflexion angle of 5° was set based on Rosenblatt et al. (2014) who found that 5° of dorsiflexion was sufficient to reduce the risk of tripping over a 5 mm unseen obstacle by a factor of 20. Based on kinematic simulations similar to those conducted by Sensinger et al. (2013), 5° of dorsiflexion was expected to yield an increase in MFC of approximately 10–15 mm. As shown in Rosenblatt et al. (2017), over a 1-year observational period, prosthesis users who reported at least one fall had an MFC that was on average 13.3 mm less than that of prosthesis users who did not fall. As such, the 5° target range of motion is expected to produce sufficient foot clearance for significant real-world fall reduction. To ensure that the ankle provides ESR comparable to that of an ESR foot, the device must adopt a neutral position (not dorsiflexed) during the stance phase of walking and not yield under the dorsiflexion torques associated with stance phase. A spring foot in series with the ankle mechanism may then serve to store and release energy. As such, the ankle will adopt two different configurations during a stride: dorsiflexed during swing phase, and a neutral position during stance phase. The ankle design is intended to operate correctly when paired with any spring foot.

To minimize mass, size, and complexity, a mechanically passive design is desirable. A passive design eliminates the need for a battery, electromechanical actuator, and any wires that may serve to increase the device size or reduce its durability/reliability. To minimize size and increase durability, mechanisms that feature surface contact between components are favorable. Surface contact between parts allows for relatively small components to sustain large loads without succumbing to stress limits. Such a requirement eliminates the possibility of using many clutch-based mechanisms that rely on point or line contact between components. Clutches, furthermore, are typically sources of wear, which can limit the ultimate lifetime of a device, and often are not silent when engaged or disengaged, which is undesirable in a prosthetic ankle. Correspondingly, clutch-based devices described in the literature have expressed limitations regarding durability, reliability, and wear as described in Williams et al. (2009), Nickel et al. (2012, 2014), and Aliukov et al. (2015).

### 2.2. Design approach using the principle of virtual work

The initial goal was to design a device that is biased toward dorsiflexion in the absence of external loads (i.e., swing phase) and will plantarflex when any substantial load is applied in stance. In order to achieve this behavior, the PDAP mechanism was conceived as a generalized coupling between a prismatic motion along the shank and the rotation of the ankle joint.

**Figure 1.**
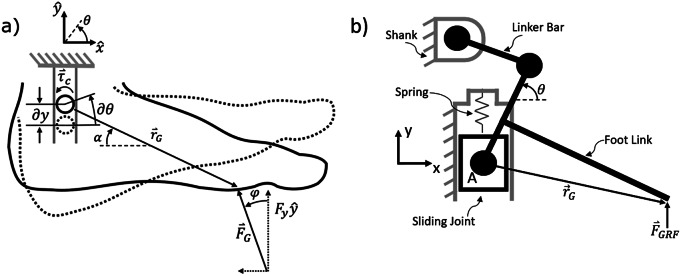
Generalized (a) and specific (b) schematic diagrams for the design of the PDAP. (a) A foot member in initial (dashed) and displaced (solid) configurations. The ankle joint is constrained to translate in the 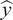 direction relative to the grounded shank. The foot has a ground reaction force, 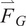 , applied near the ball of the foot. 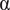 denotes the angle of 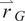 relative to 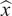 . 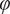 denotes the angle of 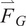 relative to 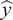 . (b) The schematic diagram of the specific PDAP linkage described in this work, including a shank (grounded), crank, linear spring, slider, and foot configured as a slider-crank mechanism. The vector, 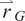 , points from the ankle joint location to the point of application of the ground reaction force, 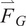 . Note that dorsiflexion is defined as a positive rotation of 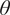 , consistent with the right-hand rule according to the shank-based coordinate frame *x*–*y.*

For the purposes of this analysis, the shank will be grounded, and the shank-attached coordinate frame depicted in Figure 1a will be adopted. For the generalized mechanism, it is assumed that a positive displacement of the foot in the 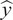 direction (relative to the shank) will yield a negative (plantarflexion) rotation of the foot about the ankle joint. The angle of the foot relative to 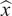 is denoted by 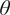 where 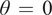 denotes the condition where the foot is perpendicular to the shank. A ground reaction force vector, 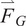 , can be applied anywhere along the bottom of the foot (and is shown near the ball of the foot in Figure 1a, which corresponds to late stance phase loading).

In the absence of a ground reaction force, the state of the mechanism will be undetermined, and so it is assumed that an internal compliant element is included that biases the device against a dorsiflexion limit stop. With such a configuration, the device will default to dorsiflexion when loads are removed, which is the desired swing behavior.

Under the assumptions given above, the design task is to select a mechanism that produces the coupling between prismatic motion and rotation that will always yield plantarflexion when a ground reaction force vector is present, regardless of its point of application on the foot. In order to derive a criterion for this design task, the Principle of Virtual Work is employed.

Whenever the line of action of 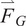 does not intersect the ankle joint, a torque is generated about the joint according to (1), where 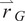 is the vector from the ankle joint to the point of application of the ground reaction force (i.e., the center of pressure).(1)

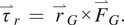

Because of the coupling described above, however, the component of 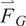 aligned with the prismatic axis of the shank will generate an additional torque about the ankle joint (i.e., in addition to the torque, 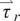 , described by above). The net force in the prismatic direction, 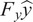 , will be comprised of both the vertical component of 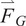 and the small negative bias force provided by the compliant element. This net force may be applied along the prismatic axis across an infinitesimal linear translation, 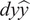 . The applied force results in a coupling torque, 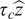 , applied about the ankle joint across an infinitesimal displacement, 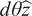 . The expression of energy flow through the mechanical system can be expressed as a statement of virtual work (2):(2)

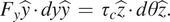



This expression may then be rearranged to provide a description of the scalar mapping between coupling torque and prismatic force on the mechanism:(3)

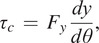

where 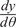 is the mechanism’s mechanical advantage. It should be noted that, in general, the mechanical advantage of the mechanism may change as a function of configuration 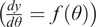 .

During stance phase, the effective point of application of the ground reaction force progresses anteriorly toward the toe, and it will eventually create an external dorsiflexion moment about the ankle joint ( 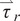 ) (Gregg et al., 2014; Prost et al., 2022). At the same time, however, the net force that is parallel with the prismatic axis ( 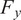 ) tends to compress the mechanism (due to the presence of a generally vertical ground reaction force). This compressive force is then transduced to a plantarflexion moment ( 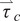 ) about the ankle joint through the mechanical advantage of the mechanism. The net torque on the foot member about the ankle joint may be determined by summing these moments.(4)

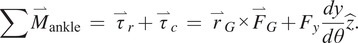



Any given design can be evaluated using biomechanical load conditions through the application of (4). However, once a design is reached, there is often a more straightforward method to analyze its performance, such as the graphical analysis presented subsequently.

At this point, several additional variables may be introduced to characterize the position of the ankle joint relative to the center of pressure and also to characterize the direction of the ground reaction force. Namely, the vector, 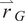 , has an angle, 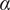 , relative to a line that is perpendicular to the shank body segment (see Figure 1a). Furthermore, the ground reaction force, 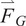 , has a direction that is characterized by the angle, 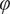 , relative to the axis of the shank (see Figure 1a). Further noting that 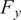 consists of the projection of 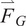 along the shank less the force of the bias spring, 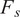 , the vector expression in (4) can be written as a scalar moment acting in the 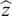 direction (5):(5)

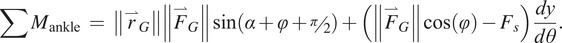



To ensure that the PDAP mechanism does not yield in the dorsiflexion direction when subject to stance phase loads, the net moment about the ankle joint must be in the plantarflexion direction ( 
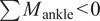
 ). Substituting (5) into this inequality constraint, simplifying, and solving for the mechanical advantage yields (6):(6)

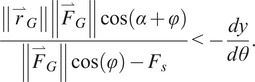



In order to use (6) to produce a general design rule for building a PDAP, several assumptions pertaining to the device design and gait can be applied. First, it is assumed that the spring force, 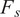 , is negligible relative to the vertical component of the ground reaction force ( 
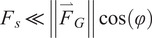
 ). If this assumption is applied to (6), the stability criterion specified in (6) becomes independent of the magnitude of the ground reaction force as seen in (7):(7)

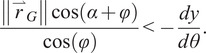



If it is further assumed that 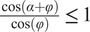 , then stability criterion in (7) simplifies further to (8), which can be used as a simple design guideline.(8)

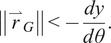



Note that 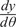 must be a negative value for (8) to be satisfied. The meaning of this result is simply that the kinematic linkage between the shank translation and the foot rotation must be such that axial compression yields plantarflexion of the foot and is merely due to sign conventions in the model. What is most important, from a design perspective, is the relative magnitudes of 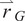 and 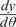 .

The assumption is that 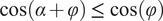 can be satisfied in a number of ways. Specifically, this assumption will be satisfied if the ankle joint is positioned low to the ground such that 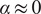 . Alternatively, this assumption will be satisfied if both 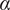 and 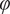 are between 0 and 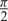 . This second assumption is generally true of human gait data during the middle and late stance phase of walking (Prost et al., 2022).

The expression in (8) indicates that if the mechanical advantage 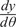 is larger in magnitude than 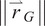 , then the ankle joint will experience a net plantarflexive torque about the ankle joint when subject to stance phase loads. Note that the criterion set forth by (8) is independent of the applied force, 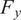 . It should be noted that in a prosthetic ankle/foot, there is a physical limit on the magnitude of 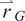 . Namely, the ground reaction force must lie on the foot ( 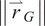 can only be approximately as large as the foot is long). Therefore, if the PDAP mechanism is designed such that the magnitude of the mechanical advantage ( 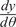 ) in stance phase is larger in magnitude than 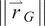 could possibly be, then the ankle will not yield in the dorsiflexion direction while subject to stance phase loads. When the ground reaction force is removed from the mechanism, the energy stored in the linear return spring is released to return the foot to a dorsiflexed position during swing phase.

Up to this point, the late stance phase geometric locking feature has been examined along with the swing phase behavior of the PDAP. However, the early stance heel strike behavior has yet to be discussed. During heel strike, (4) can be used to analyze the PDAP’s behavior. At heel strike, the ground reaction force vector, 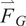 , will be located near the heel and will be pointed in a generally vertical direction. As such, 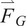 , will point very closely to the ankle joint and the 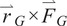 term in (4) will be small in magnitude. However, due to the generally vertical nature of 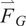 at heel strike, the 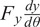 term in (4) will be large in magnitude. Generally speaking, at heel strike, the 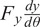 term will tend to dominate (4) due to the large magnitude of 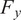 , and the net ankle moment will be negative, causing a plantarflexive motion of the ankle joint. This plantarflexive motion of the ankle joint will occur at heel strike as long as the ground reaction force vector points either posterior to the ankle joint center (yielding a negative value of 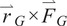 ) or near the ankle joint center and anterior to the joint center (yielding a small, positive magnitude of 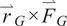 ).

In the event that a user executes a fore- or mid-foot strike while walking with the PDAP, its behavior will still be determined by the location and direction of the ground reaction force vector. As long as 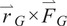 is a negative value, the ankle will always move to full plantarflexion when loaded. Furthermore, the dorsiflexed configuration of the device in swing makes it difficult to achieve forefoot strikes when walking on level ground. When encountering inclines, however, it is possible to implement this theory in a way that prevents the ankle from plantarflexing after a forefoot strike (due to a sufficiently large value of 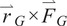 ). In this case, the dorsiflexed configuration has the potential to better conform to the incline.

### 2.3. Implementation using a slider-crank mechanism

The particular PDAP mechanism settled upon by the authors is a preloaded slider-crank mechanism with a limited range of motion. The shank serves as the grounded link and contains the sliding interface for the prismatic joint. The foot serves as the connecting rod, connecting the slider and crank as pictured in Figure 1b. The linear spring preloads the slider relative to the shank in its extended position.

Recall that the goal of the PDAP is to dorsiflex the ankle during swing phase and maintain a neutral position during stance phase. Under the assumption that there is no external loading of the device during swing phase, the ankle will dorsiflex through the action of the internal spring element which forces the slider away from the shank (Figure 1b). At heel strike, the ground reaction force tends to force the slider toward the shank, and the foot plantarflexes until the slider contacts a mechanical limit stop. When the limit stop is reached, the foot is in a neutral configuration. The foot then remains in this neutral configuration for all (realistic) ground reaction force vectors present for the remainder of stance, and only returns to the dorsiflexed position when the load is removed.

**Figure 2.**
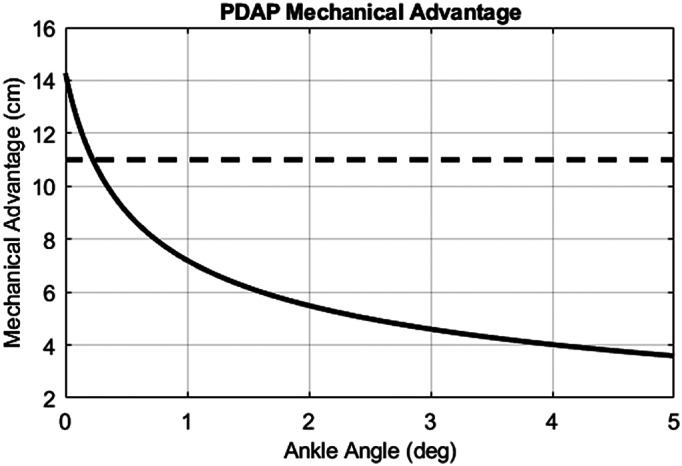
PDAP mechanical advantage magnitude, ||*dy/dθ*||, plotted against ankle angle, 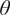 , (solid black line). The physical limit of 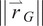 is plotted as a dashed horizontal line. Note that when the ankle is at 0°, the mechanical advantage is larger than the physical limit of 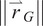 , thereby ensuring stance phase stability.

To ensure that the PDAP provides the desired stance phase-locking behavior, the magnitude of the mechanical advantage of the PDAP linkage, 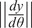 , is plotted as a function of ankle angle in Figure 2. The mechanical advantage term, 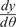 , is derived from the kinematics of a standard slider-crank mechanism. The figure also shows a horizontal dashed line which serves to represent the physical limit of 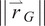 based on realistic prosthetic feet. As seen in Figure 2, when the ankle angle is at 0°, the mechanical advantage is larger in magnitude than the physical limit of 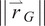 (shown as a dashed line). This mechanical advantage ensures the stance phase-locking behavior based on (8).

### 2.4. Interpretation using the instantaneous center of rotation

**Figure 3.**
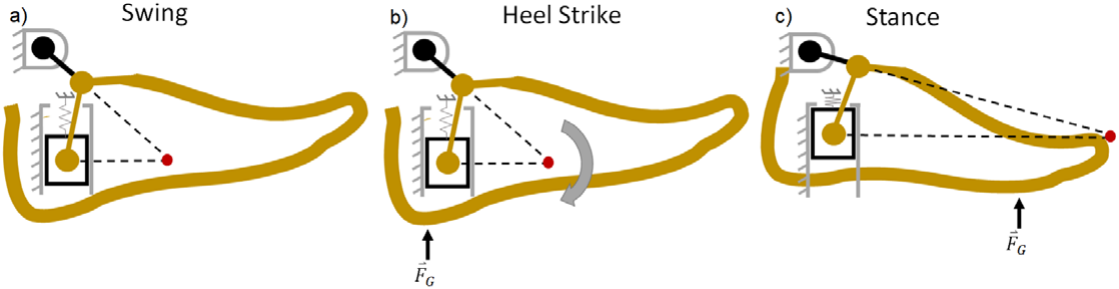
Diagrams of the functional states of the passive dorsiflexing ankle prosthesis (PDAP). (a) The configuration of the PDAP in swing (dorsiflexed). (b) The PDAP at the instant of heel strike, when an external load produces a plantarflexive torque to return the ankle to a neutral position. (c) The PDAP during stance, when the instant center of rotation has moved beyond the toe such that all attainable external loads still yield a plantarflexive torque. The mechanism’s ICR is depicted as a red dot.

Although the function of the PDAP mechanism can be fully described using the virtual work method developed above, it is also possible to analyze its behavior using graphical methods. In this slider crank arrangement, the foot rotates about an instantaneous center of rotation (ICR) that moves as a function of the mechanism’s configuration. This ICR can be found graphically via the intersection of two lines (one in-line with the crank and the other which is perpendicular to the prismatic axis of the slider) as shown in Figure 3. The figure depicts the PDAP mechanism in both the swing phase and stance phase configurations, as well as at the moment of heel strike, along with the location of the ICR (shown in red).

When the foot is under no load, the linear return spring is at its preloaded length, and the foot adopts a dorsiflexed angle relative to the shank (see Swing in Figure 3). When a substantially vertical load is applied to the foot at heel strike, the spring is compressed, and the foot plantarflexes until a mechanical limit stop is reached (at a neutral ankle angle). Additionally, during this motion, the ICR moves to a point beyond the toe of the prosthesis (see Stance in Figure 3). Due to the distal position of the ICR in stance, any load applied to the foot will be posterior to the ICR and will cause a plantarflexion torque. Because the ankle is against a plantarflexion limit stop in this configuration, a geometric lock is achieved such that any vertical loads applied to the foot will not move the PDAP mechanism. Due to this geometric lock, a composite energy storage and release foot in series with the PDAP mechanism is able to store and release energy during stance phase. All that is required to regain dorsiflexion (unlock the mechanism) is to unload the ankle, which occurs naturally at the initiation of swing phase. When the ankle joint is unloaded, the spring releases its stored energy and returns the PDAP mechanism to its dorsiflexed swing phase configuration.

In the PDAP mechanism, the foot both translates and rotates relative to the shank. During swing phase, the effective leg length increases while the foot simultaneously dorsiflexes. These two motions have competing effects on MFC that must be carefully considered during the design process. The lengthening of the leg due to the prismatic motion of the foot relative to the shank tends to decrease MFC while the rotation of the foot relative to the shank in the dorsiflexion direction tends to increase MFC. The PDAP mechanism has been carefully designed to achieve a significant net increase in MFC through linkage optimization and kinematic simulations (similar to the simulations performed by Sensinger et al. (2013)). To ensure that there is a net increase in MFC, it is advantageous for the ICR to remain proximal to the shank across the majority of the ankle joint’s range of motion. In this way, the foot primarily rotates without significant axial translation. However, in order to achieve the geometric locking condition necessary for the PDAP’s stance phase behavior, the ICR must quickly move past the toe as the ankle joint approaches its limit stop. To gain a more intuitive understanding of this phenomenon, consider a mechanism in which the ICR is always located very far past the toe. In this mechanism, the foot would have to translate substantially relative to the shank to achieve a desired angular rotation. Consequently, to achieve substantial angular rotation with minimal axial translation, it is advantageous for the ICR to be proximal to the shank for most of the ankle joint’s range of motion.

### 2.5. Biomechanical operation

A diagram of the PDAP is shown in different phases of gait in Figure 3. During early stance, the ankle undergoes a plantarflexion motion until the device reaches its limit stop. During this phase of gait, the linear spring serves to cushion the heel strike event, and energy from this impact is stored as elastic potential energy. During middle stance, the ground reaction force is located along the keel of the foot. However, the ankle is in its geometrically locked configuration and cannot yield under these loads. During terminal stance, the ankle remains in its geometrically locked configuration as energy is stored and subsequently released by the series spring foot. When the ground reaction force is removed as the user enters swing phase, the energy stored in the linear spring is released to dorsiflex the PDAP device during swing phase. The device is now configured for the heel strike of the subsequent stride.

Although the PDAP was primarily designed for level ground walking, other activities of daily living were also considered during the design phase. It is expected to behave comparably to ESR feet during sloped walking. Furthermore, during standing, the PDAP is expected to adopt a neutral configuration (due to the presence of a vertical ground reaction force) and provide full standing support.

### 2.6. Hardware implementation

**Figure 4.**
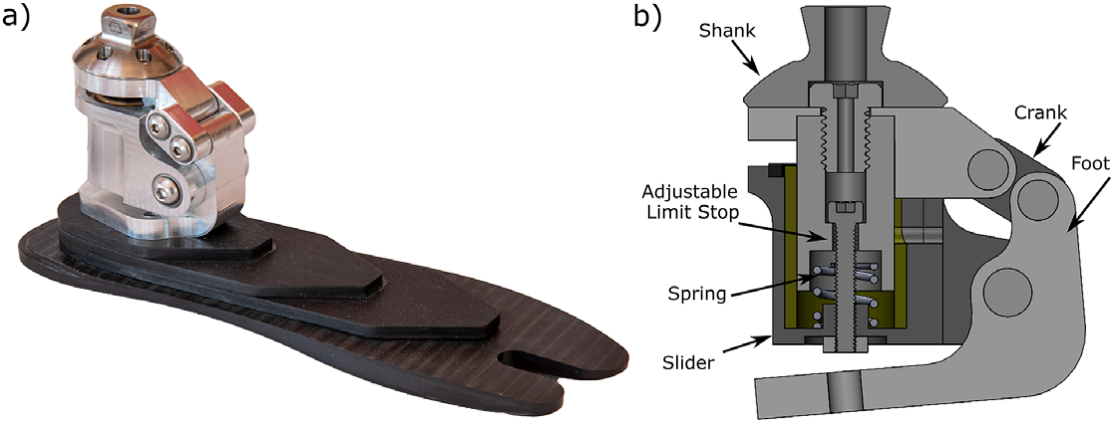
Depictions of the PDAP hardware prototype. (a) A photograph of the PDAP as constructed and assembled with the compliant low-profile foot. (b) A sagittal plane cutaway of the PDAP CAD configured for the swing phase (dorsiflexed) configuration. Walking direction is to the right.

A prototype of the PDAP was constructed according to the design presented above and is shown in Figure 4a. The PDAP mechanism is paired with a custom-compliant low-profile foot designed using the methods described in Bartlett et al. (2022b). The foot was selected to have a comparable stiffness to prosthetic feet prescribed at the K3 activity level (Turner et al., 2022). The device (including compliant foot) has a mass of 620 g and a build height of 8.5 cm, which is comparable to many commercially available ESR feet (Bartlett et al., 2022b). The PDAP dorsiflexes 5° and displaces 5 mm relative to the shank during swing phase. The device has been designed to fit within a commercial cosmetic foot cover. A computer-generated sagittal plane cutaway of the device is shown in Figure 4b. The sliding link is implemented as a shaft sliding within a linear bearing, while the rotary joints are all implemented with doubly supported shafts. The linear spring element is a coil spring. It should be noted that every component within the PDAP mechanism experiences surface contact when interacting with other components. This surface contact helps to minimize the device size and mass while maintaining strength. The PDAP device also includes a feature for locking the device in the neutral position and disabling its swing phase dorsiflexion functionality. This functionality is achieved through an adjustable dorsiflexion limit stop which is implemented as a screw within the prismatic joint. To prevent swing phase dorsiflexion, this limit stop can be adjusted so that the linear degree of freedom has zero range of motion. This feature was used during the assessments described subsequently.

## 3. Assessment

The PDAP was assessed in level ground walking experiments with a single unilateral transtibial prosthesis user (participant mass: 90 kg). Ethical approval to perform these experiments was granted by the Northeastern University Institutional Review Board, and written informed consent was obtained from the participant prior to the assessment.

### 3.1. Experimental protocol

**Figure 5.**
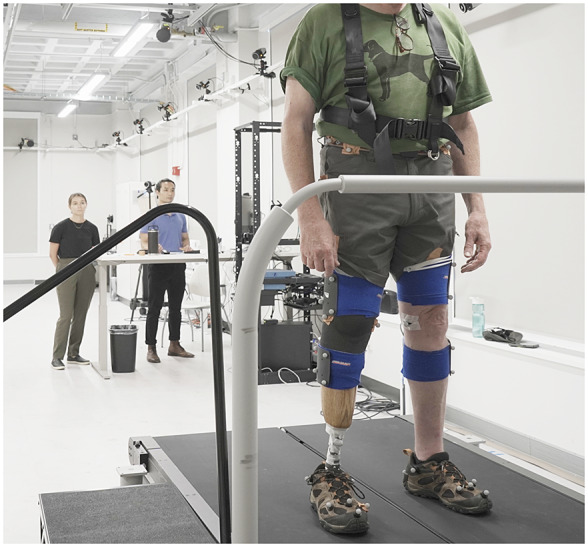
Study participant wearing the PDAP device during level ground walking assessment.

The experiment consisted of a level ground walking trial conducted on a split-belt force-instrumented treadmill (Bertec). The walking trial consisted of walking at a speed of 1 m/s for 90 s (Figure 5). The participant walked while wearing shoes in three different prosthetic device conditions: (1) daily use ESR foot (size 27 Fillauer AllPro), (2) PDAP in a fully operational mode (unlocked PDAP), and (3) the PDAP with the swing phase dorsiflexion feature disabled (locked PDAP). The PDAP was fit to the participant and aligned by a certified prosthetist. During the experiments, ground reaction force data were collected under each foot at 1,200 Hz, and lower-body kinematics were recorded at 300 Hz via a synchronized motion capture system (Qualisys).

### 3.2. Biomechanical outcome measures

The ankle joint angle and ankle torque were calculated using the final 40 s of the collected kinematic and kinetic data of each walking trial (to allow the subject to reach a steady-state gait) and was used to characterize the ankle behavior during each of the prosthetic conditions. Strides in which the participant stepped across the centerline of the split-belt treadmill were excluded from analysis. At least 25 strides were collected for each prosthetic condition. Ankle kinematic and kinetic data were filtered using a forward-reverse 4th order lowpass Butterworth filter with a 10 Hz cutoff frequency.

In order to characterize the ankle behavior in each of the prosthetic conditions, ankle angle, ankle torque, energy return, and MFC were all calculated. Sagittal plane ankle angle was calculated from the projection of the skeletal model vectors of the shank and foot onto the sagittal plane. The shank vector was defined as the vector between the ankle joint marker and a marker on the lateral epicondyle marker. The foot vector was defined as the vector between the ankle marker and a marker placed on the second metatarsal head. Sagittal plane ankle torque was calculated via a cross product between a vector pointing from the ankle joint (the location of which was approximated as the location of the marker placed on the lateral malleolus) to the center of pressure and the ground reaction force vector. Ankle joint sign conventions used in the data analysis are consistent with those of Figure 1. MFC was calculated by measuring the kinematic trajectory of a marker placed above the equivalent location of the second metatarsal head on the prosthetic device, using the contralateral foot as a reference. Similar measures of MFC have been employed in other works (Rosenblatt et al., 2014; Lamers et al., 2018; Riveras et al., 2020; Bartlett et al., 2021). Although this method of measuring MFC only captures the location of a single point on the foot (and not the minimum height of any point on the foot), it is a simple approximation of the true MFC. Energy return was quantified as the percent energy returned by the prosthesis at terminal stance (a measure of energetic efficiency of the ankle and foot). To compute this energy return percentage, ankle power was computed as the product of ankle angular velocity and torque. Ankle power was then integrated for each stride with respect to time to yield the energy stored in the prosthesis as a function of time. This energy was then stride normalized and averaged across strides. The percent energy returned at terminal stance (after the instant of peak energy storage/dissipation) was then computed. As such, if a device exhibited a 25% energy return, then 75% of the energy stored in the device during stance would have been dissipated. Finally, data was time-normalized in terms of percentage of stride in order to generate plots parameterized by gait phase.

In the locked PDAP condition, the PDAP mechanism cannot move during stance or swing phase, and therefore, it behaves solely as a spring-like ankle/foot complex (comparable to standard ESR feet). By comparing the locked and unlocked PDAP conditions, the function of the PDAP mechanism can be directly assessed while controlling for other factors associated with the prosthetic device such as mass or foot stiffness. Consequently, the locked PDAP condition serves as an experimental control that allows for a direct assessment of the PDAP’s swing dorsiflexion functionality.

## 4. Results

**Figure 6.**
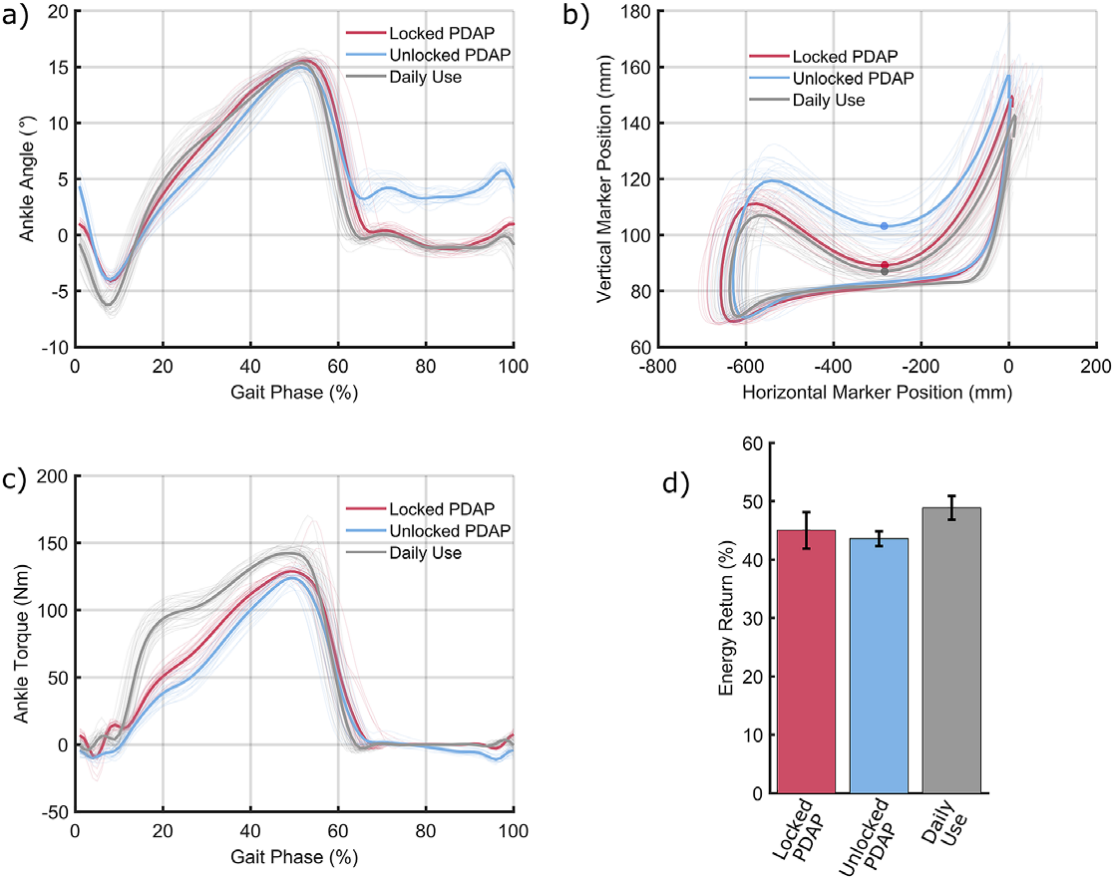
Biomechanical results from the level walking assessment. (a) Ankle angle plotted as a function of stride for the unlocked (fully functional) PDAP (blue), the locked PDAP (red), and the participant’s daily use prosthesis (gray). (b) Foot clearance trajectory calculated by tracking the trajectory of a toe marker in the lab-based reference frame. The unlocked (fully functional) PDAP is plotted in blue while the locked PDAP is plotted in red and the daily use device is plotted in gray. Instances of minimum foot clearance are plotted as solid dots. Heel strikes occur in the top right corner of the plotted trajectories, and the trajectories flow in a clockwise direction over the course of a stride. (c) Ankle torque plotted as a function of percentage of stride for the locked PDAP (red), unlocked PDAP (blue), and the daily use device (gray). (d) Energy return percentage at terminal stance for the locked PDAP (red), unlocked PDAP (blue), and daily use prosthesis (gray).

The ankle joint kinematics of the PDAP in the locked (red), unlocked (blue), and daily use (gray) prosthesis conditions are shown in Figure 6a. In Figure 6a (as well as Figure 6c), the kinematic and kinetic outcome variables are stride normalized, where a stride is defined as the time period from one heel strike to the subsequent heel strike of the same leg. Stance behavior (10–65% of stride) is similar for all conditions. However, in swing phase (65–100% of stride), the unlocked PDAP exhibits a different ankle angle (approximately 5° of dorsiflexion) when compared to the other prosthetic conditions. It should be noted that in Figure 6a, the measured ankle angle incorporates the motion of the PDAP mechanism as well as the deformation of the compliant foot, cosmetic foot cover, and shoe.

The effect of this swing phase dorsiflexion on MFC is examined in Figure 6b in which the trajectory of a marker placed on the toe is tracked in the laboratory-based reference frame. In Figure 6b, the positive *X* direction indicates the forward walking direction. Trajectories in Figure 6b begin with heel strike in the top right of the plot and travel in a clockwise direction over the course of a stride. Swing phase can be identified as the region in the plot when the horizontal position of the toe marker is increasing. The solid dots marked on the figure indicate the instant of MFC. As shown in Figure 6b, the unlocked PDAP achieves 13 mm more foot clearance than the locked PDAP and 15 mm more foot clearance than the daily-use device. Specifically, the vertical toe marker positions at the instant of MFC were 88 ± 2.0, 102 ± 4.0, and 86 ± 2.7 mm for the locked PDAP, unlocked PDAP, and daily-use devices, respectively. Two separate one-tailed *t*-tests were performed (along with a Bonferroni correction) to assess the statistical significance of the increased foot clearance achieved by the unlocked PDAP when compared to the other two prosthesis conditions. Using an alpha value of 0.05, the increase in foot clearance achieved by the unlocked PDAP was shown to be statistically significant (*p* < 10^–7^). To assess the degree to which compensatory actions at intact joints contributed to the observed differences in MFC, the ankle marker vertical positions and global orientations of the shank body segment were measured at the instant of MFC. This information captures the cumulative effects of any upstream compensations in the lower body kinematic chain (hip position, hip angle, or knee angle differences). The mean ankle marker vertical positions varied by 2 mm across all prosthetic conditions (and the locked and unlocked marker positions had a difference of 1 mm). Furthermore, the mean orientation of the shank body segment varied by 0.2 deg across all prosthesis conditions. To assess the influence of the PDAP mechanism on stance phase kinetics, the ankle torque during a stride was calculated and is shown in Figure 6c. In addition to ankle joint angle and torque, the energy return percentage at terminal stance was also computed for all three prosthesis conditions and is shown in Figure 6d. The energy return percentages at terminal stance were 45 ± 3, 44 ± 1, and 49 ± 2 for the locked PDAP, unlocked PDAP, and daily-use devices, respectively. As seen in Figure 6c, the stance phase torques are very similar between the locked and unlocked PDAP conditions. The torque associated with the daily-use device is slightly higher than the PDAP condition and has a faster rise time. Figure 6d shows that the energy return percentage is comparable for all three prosthesis conditions.

## 5. Discussion

### 5.1. Interpretation of results

As seen in Figure 6a, the heel strike behaviors (0–10% of stride) of the three prostheses differ slightly. The daily use device and the locked PDAP both undergo qualitatively similar amounts of plantarflexion motion during heel strike which may be due in part to compliance of the cosmetic foot cover and shoe. The unlocked PDAP shows more plantarflexion at heel strike than the other two conditions due to the initial motion of the mechanism, along with the same cosmetic foot cover and shoe compliance.

During stance phase, the three ankles exhibit very similar ankle joint kinematics. When comparing the locked and unlocked PDAP joint angles, the similar behavior between these two conditions indicates that, in the unlocked condition, the ankle is not yielding during stance phase and that the geometric lock of the PDAP is operating as intended. When comparing the PDAP (both locked and unlocked) behavior to that of the daily use device, the similar behavior in stance phase indicates that the low-profile foot in series with the PDAP is behaving similarly to the commercial ESR foot worn by the participant. However, the ESR foot does exhibit a significant plantarflexion motion during heel strike due to its compliant heel component. Importantly, during the swing phase of gait, both the locked PDAP and the daily use device return to their neutral configurations (0°) while the unlocked PDAP adopts a dorsiflexed ankle angle of approximately 5°. This indicates that the passive dorsiflexing functionality of the PDAP is operating as designed.

The dorsiflexed position of the unlocked PDAP during swing phase (seen in Figure 6a) is intended to increase swing phase foot clearance of the device in an effort to reduce the risk of stumbles and falls. This foot clearance is shown via dots in Figure 6b which highlight the instant of swing phase MFC. As seen in Figure 6b (and verified with the previously described statistical analysis), the unlocked PDAP achieves a significant increase in MFC when compared to either the locked PDAP or the daily use prosthesis. Furthermore, due to the very small range of ankle marker vertical heights and shank orientations observed at the instant of MFC, these changes in MFC across prosthesis conditions are due almost exclusively to differences in the ankle joint behaviors as opposed to compensatory actions adopted by the user.

To ensure that the increase in MFC obtained by the unlocked PDAP is not at the cost of stance phase functionality, the ankle torque and energy return of the three prosthesis conditions were examined and are shown in Figure 6c,d, respectively. As shown in Figure 6c, the ankle torques in the locked and unlocked PDAP conditions are very similar, indicating that the operation of the PDAP mechanism does not substantially impact ankle torque during stance phase. Furthermore, the general shape and magnitudes of the torque curves in the PDAP conditions and the daily use prosthesis conditions are qualitatively similar, indicating that the low-profile foot in series with the PDAP mechanism has a qualitatively similar behavior to that of the ESR daily use device. As previously mentioned, when wearing the daily use device, the stance torques are slightly higher than when wearing the PDAP. This is likely due to compliance differences between feet. By inspection of Figure 6a,c, the ESR daily use device requires more ankle torque to achieve comparable angular displacements to that of the PDAP, indicating that it is a stiffer foot. However, differences in foot behavior were experimentally controlled for through the comparison of the locked and unlocked PDAP conditions. The energy return properties of the prosthetic devices were also examined as seen in Figure 6d. It should be noted that the energy return efficiencies of the locked and unlocked PDAP conditions are very similar. This similarity in energy return indicates that the operation of the PDAP mechanism does not negatively impact the stance phase energy return properties of the compliant foot with which it is paired. Moreover, the energy return percentage of the low-profile foot (as characterized by the energy return percentage in the locked PDAP condition) is similar, although slightly less than that of the ESR daily use foot.

### 5.2. Device design

As can be seen in Figure 6a,c,d, the PDAP mechanism provides swing phase dorsiflexion without compromising stance phase behavior. The ankle mechanism adopts a dorsiflexed position when under no load (during swing phase) and adopts a neutral configuration during stance phase. Furthermore, when subject to stance phase loads, the PDAP mechanism does not yield in the dorsiflexion direction, and instead, the series spring foot stores and releases energy comparably to conventional ESR feet. It should be noted that although the PDAP is paired with a custom-compliant low-profile foot in this work, the PDAP device could be paired with other compliant feet without loss of functionality. One other component in the PDAP that deserves careful attention is the linear spring element. The purpose of this spring is to both provide impact absorption during heel strike and to provide the energy necessary to dorsiflex the ankle during swing phase. In this way, the linear spring element is used to recycle energy from heel strike to dorsiflex the ankle during the subsequent swing phase. The stiffness of this spring has important implications on the function of the PDAP during gait. If the spring is too stiff (or under too much preload), then the spring force may not be negligible compared to the axial component of the user’s ground reaction force. In this case, the spring may have a deleterious effect on the stance phase locking functionality of the PDAP. However, if the spring is too compliant (or not subject to enough preload), then the ankle may plantarflex very rapidly at heel strike. Rapid heel strike behavior of a prosthetic foot is sometimes referred to as “foot slap” and is generally disliked by prosthesis users (Shepherd and Rouse, 2020). As such, the spring should be chosen so that it is compliant enough to not have a negative effect on the geometric locking function of the PDAP but it should be stiff enough to avoid “foot slap.” This spring stiffness (180 N/cm) was determined by trial-and-error in the device presented in this work.

The PDAP adopts different configurations in the swing and stance phases of gait. It does this by leveraging the user’s loading of the device to automatically change its configuration and behavior. In this way, whenever the user’s weight is on the device, it behaves like an ESR foot (via the geometric lock of the mechanism), and whenever the user’s weight is removed from the device, the ankle joint adopts a dorsiflexed position. The intent is that this simple mechanical logic allows the device to operate appropriately across a large range of biomechanical tasks such as walking, standing, or stair ambulation. However, these other biomechanical tasks have yet to be formally assessed.

In Section 2, two different methods are used to analyze the behavior of the PDAP: (1) a method using the Principle of Virtual Work, and (2) the method of instant centers of rotation. These two methods are both valid analysis approaches but may have strengths or weaknesses depending on what a designer seeks to gain from their analysis.

One strength of the method of virtual work is that the analysis is mechanism agnostic. The method (as employed in this work) only assumes that there is a kinematic coupling between an axial degree of freedom, 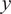 , and a rotational degree of freedom, 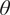 , that can be described via a mechanical advantage, 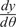 . A stance stability criterion is then developed that sets a bound for 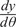 when the ankle is in its neutral position (8). This approach may lend itself to numerical methods for determining viable mechanisms.

Although the kinematic coupling between the axial and rotational degrees of freedom in this work is a slider-crank linkage, other mechanical mechanisms may also satisfy this stability criterion. It should be noted that, in the PDAP mechanism, the magnitude of 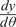 becomes very large (larger than the physical limit of 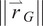 ) as the ankle approaches its neutral position. This occurs because the mechanism is nearing a singular position when the ankle reaches a neutral position. Specifically, if the crank were to reach the configuration in which it was perpendicular to the prismatic axis of the slider, the PDAP mechanism would be in a singular configuration, and the magnitude of 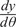 would be infinite.

The method of instant centers of rotation is arguably a more useful and practical way to analyze the specific PDAP mechanism employed in this work. This analysis method is graphical in nature and allows a designer to analyze the stance phase stability characteristics of the mechanism quickly and visually. Furthermore, the instant center of rotation method provides a geometric and physical intuition to the mechanism design process, allowing for easier understanding of the device’s function. In order to employ this method, however, certain assumptions about the mechanism (such as the mechanism type or linkage topology) must be made a priori, resulting in a trial-and-error design process.

The method of instant centers of rotation can also be used to understand how the PDAP may function during gait activities other than level ground walking. For the PDAP to achieve its geometric lock, it must first undergo a plantarflexive motion upon contact with the ground. For such a plantarflexive motion to occur, a ground reaction force must be posterior to the ICR. This is the case during a heel strike event in which the ground reaction force is mostly vertical and is located at the heel. This posterior location of the ground reaction force is also generally true during standing as well as slope descent. During slope ascent, some individuals tend to initially contact the ground with their forefoot, in which case the ground reaction force may be anterior to the ICR (Lamers et al., 2019). In this case, the PDAP would not undergo plantarflexion and instead would remain in a dorsiflexed position during the stride. In this case, however, the nominal position of the PDAP mechanism would be dorsiflexed (by 5°), which allows for some degree of adaptation to the sloped terrain (Shepherd et al., 2020; Bartlett et al., 2021). Nonetheless, the PDAP’s functionality should be experimentally assessed across various terrains and activities to fully characterize its behavior.

### 5.3. Clinical relevance

As seen in Figure 6b, the swing phase dorsiflexion behavior of the PDAP translates to an increase in MFC during the swing phase of gait. This figure shows that when the PDAP’s swing phase dorsiflexion feature is active, MFC is increased by 13 mm. This improvement in MFC is approximately the same as the MFC improvement obtained by the Proprio Foot’s active swing dorsiflexion feature (11.4 mm) (Rosenblatt et al., 2014). However, the PDAP weighs approximately 40% that of the Proprio foot and is completely passive. The Proprio foot does, however, provide more functionality than purely swing phase dorsiflexion (such as continuous slope adaptation). Based on the results reported by Rosenblatt et al. (2014)), the improvements in MFC provided by the PDAP are sufficient to reduce the risk of tripping over a 5 mm unseen obstacle by over a factor of 20. Additionally, the 13 mm increase in MFC obtained by the PDAP in the current study is comparable to the 13.3 mm difference in MFC observed between fallers and nonfallers in a 1-year real-world study (Rosenblatt et al., 2017). As such, the MFC increase provided by the PDAP may be sufficient to decrease the rate of falls experienced by prosthesis users in the real world.

The PDAP provides swing phase foot clearance by dorsiflexing the ankle when unloaded. Consequently, for a given amount of dorsiflexion (5°), the PDAP will provide more foot clearance for a user with a long foot relative to an individual with a short foot. The user-tested in this work used a size 27 cm foot. Commercial prosthetic feet made for adults typically range in size from 22 to 30 cm (Doty, 2020). Based on kinematic simulations similar to the ones performed by Sensinger et al. (2013), it is estimated that the MFC provided by the PDAP will vary by 26% across this full range of adult foot sizes. As such, the PDAP should provide an increase in MFC (relative to a foot without swing phase dorsiflexion) between 11 and 15 mm.

In addition to lowering the risk of falls, increasing MFC has many other potential health benefits for prosthesis users as described in Lechler and Kristjansson (2018). One possible benefit of increasing MFC may be to reduce back pain experienced by prosthesis users. A common method employed by prosthetists to combat the high fall rate seen in their patients is to configure the prosthetic leg to be shorter than the sound side, thereby increasing the clearance between the swing leg and ground during swing phase (Lechler and Kristjansson, 2018). In a study of 113 prosthesis users, 70% were found to have significant leg length discrepancies when standing (Friberg, 1984). A separate study of 47 prosthesis users found that 57% of participants exhibited limb length discrepancies with the prosthetic limb being shorter than the intact limb (Gaunaurd et al., 2011). Although this limb length discrepancy improves swing phase ground clearance, it may also lead to low back pain (Biering-SØRensen, 1984; Mahar et al., 1985; Defrin et al., 2005). The prevalence of low back pain within prosthesis users is estimated between 50 and 90% (Smith et al., 1999; Ephraim et al., 2005; Kulkarni et al., 2005; Kusljugić et al., 2006; Hammarlund et al., 2011; Foote et al., 2015; Sivapuratharasu et al., 2019). The improvements in MFC provided by the PDAP may allow for clinicians to configure prosthetic limbs without leg length discrepancy (by changing prosthetic alignment) and hopefully combat the high incidence of back pain. The reduction in limb length discrepancies may also help to reduce swing phase compensatory actions often adopted by prosthesis users to increase swing phase ground clearance (Kaufman et al., 1996, Sensinger et al., 2013. These compensatory actions and associated kinematic asymmetries have been linked to an increase in the long-term risk of developing osteoarthritis (Hurley et al., 1990; Lemaire and Fisher, 1994; Lechler and Kristjansson, 2018). As such, the increased MFC provided by the PDAP may result in myriad health benefits to prosthesis users, although a direct assessment of the PDAP’s effects on such long-term outcomes has yet to be performed. Importantly, the PDAP is able to provide an increase in MFC without sacrificing the stance phase stability or ESR properties of the elastic foot with which it is paired.

## 6. Conclusion

This article presents the design and assessment of the PDAP. The PDAP uses a novel mechanism to provide the following behaviors during walking: impact absorption at heel strike, ESR behavior during stance (with the stiffness-like behavior occurring about a neutral position), and swing phase dorsiflexion. The design theory underlying this mechanism is presented and then implemented in a wearable prosthetic ankle device. The PDAP was then assessed on a single transtibial prosthesis user during level ground walking and compared to the behavior of both the participant’s daily-use prosthesis and a locked version of the PDAP (with novel behaviors disabled). The PDAP was shown to increase swing phase MFC by 13 mm while not impacting the stance phase behavior of the foot with which it was paired. The PDAP shows promise as a lightweight and passive prosthetic device that may be able to reduce the risk of falls for prosthesis users. Due to the preservation of ESR, the clinical benefits of that functionality are not sacrificed to obtain an increase in MFC with the PDAP.

## Data Availability

Data will be made available upon the author request.
